# ^19^F NMR Untersuchung des Konformationsaustauschs mehrerer Zustände im synthetischen Neomycin‐bindenden Riboschalter

**DOI:** 10.1002/ange.202218064

**Published:** 2023-04-28

**Authors:** Jan H. Overbeck, Jennifer Vögele, Felix Nussbaumer, Elke Duchardt‐Ferner, Christoph Kreutz, Jens Wöhnert, Remco Sprangers

**Affiliations:** ^1^ Department of Biophysics I Regensburg Center for Biochemistry University of Regensburg Universitätsstrasse 31 93051 Regensburg Deutschland; ^2^ Institute for Molecular Biosciences Goethe-University Frankfurt Max-von-Laue-Str. 9 60438 Frankfurt/M. Deutschland; ^3^ Institute of Organic Chemistry and Center for Molecular Biosciences Innsbruck (CMBI) University of Innsbruck Innsbruck Österreich

**Keywords:** Aminoglykoside, ^19^F NMR, NMR-Spektroskopie, RNA-Dynamik

## Abstract

Der synthetische Neomycin‐bindende Riboschalter interagiert mit seinem Liganden Neomycin sowie mit den verwandten Antibiotika Ribostamycin und Paromomycin. Die Bindung dieser Aminoglykoside induziert sehr ähnliche Grundzustandsstrukturen in der RNA, allerdings kann nur Neomycin die Initiierung der Translation effizient unterdrücken. Der molekulare Ursprung dieser Unterschiede wurde auf Unterschiede in der Dynamik der Ligand‐Riboschalter‐Komplexe zurückgeführt. In diesem Artikel kombinieren wir fünf komplementäre fluorbasierte NMR‐Methoden, um die Dynamik der drei Riboschalter‐Komplexe im Sekunden‐ bis Mikrosekundenbereich genau zu quantifizieren. Unsere Daten offenbaren komplexe Austauschprozesse mit bis zu vier strukturell unterschiedlichen Zuständen. Wir interpretieren unsere Ergebnisse in einem Modell, das ein Zusammenspiel zwischen verschiedenen chemischen Gruppen in den Antibiotika und spezifischen Basen im Riboschalter zeigt. Allgemeiner unterstreichen unsere Daten das Potenzial von ^19^F NMR‐Methoden, komplexe Austauschprozesse mit mehreren angeregten Zuständen zu charakterisieren.

## Graphical abstract

Molecular motions are tightly coupled with function. Here we use a unique combination of five different fluorine NMR relaxation methods to reveal the dynamics in three structurally similar riboswitch:ligand complexes. Our data displays unexpectedly complex exchange topologies with up to four structurally different states. Importantly, the associated kinetic and thermodynamics parameters could be accurately determined.

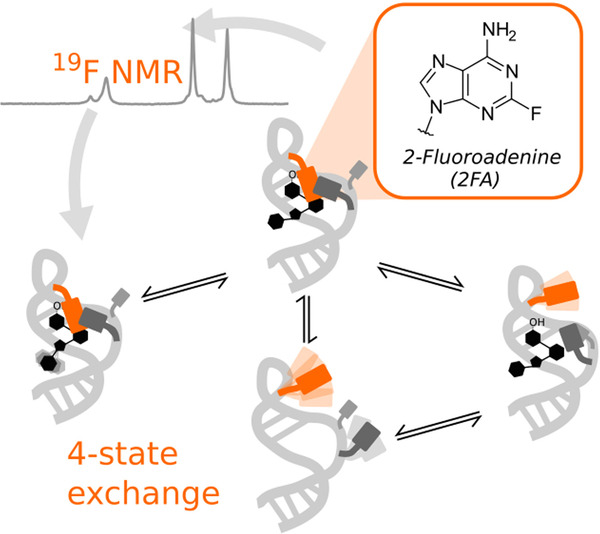

## Einleitung

In den letzten Jahrzehnten haben experimentelle und rechnergestützte Fortschritte einen umfassenden Einblick in die Struktur einer Vielzahl von Biomolekülen geliefert. Gleichzeitig hinkt unser Wissen darüber, wie diese Biomoleküle im Laufe der Zeit ihre Form verändern, deutlich hinterher,[^1^, ^2^, ^3^] insbesondere wenn mehr als zwei strukturell unterschiedliche Zustände gleichzeitig eingenommen werden können.[^4^, ^5^, ^6^, ^7^, ^8^, ^9^, ^10^, ^11^, ^12^, ^13^] Es ist jedoch klar, dass diese dynamischen Prozesse von zentraler Bedeutung sind, z. B. für die enzymatische Funktion, molekulare Erkennung, allosterische Regulierung und biomolekulare Stabilität. Die NMR‐Spektroskopie ist in der Lage Bewegungen von Biomolekülen sowohl in Proteinen als auch in RNA genau zu quantifizieren,[^14^, ^15^, ^16^, ^17^, ^18^, ^19^] und es wurden spezielle Experimente entwickelt, die empfindlich für Bewegungen auf verschiedenen Zeitskalen sind.[^2^, ^20^]

**Figure 1:**
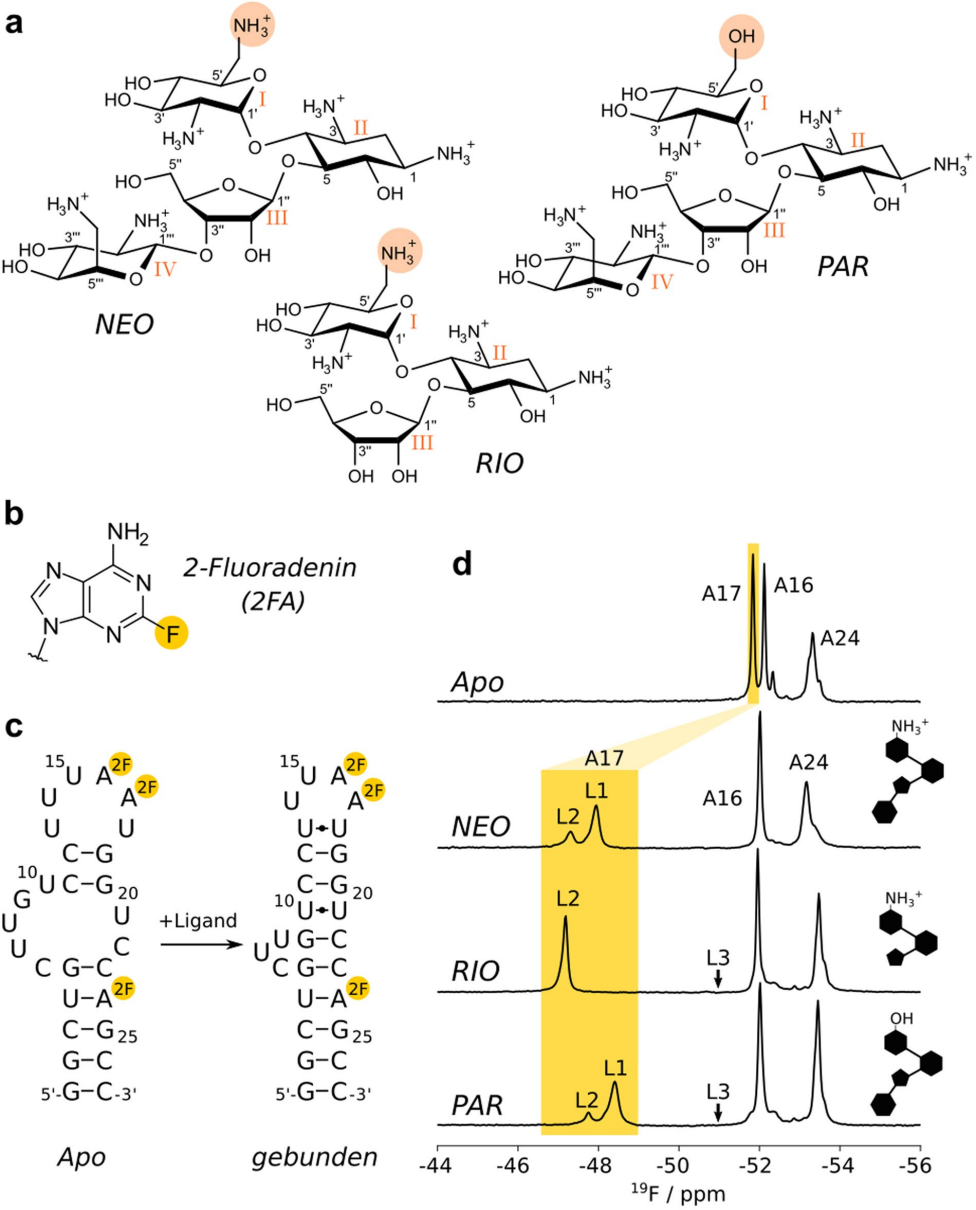
Strukturen von Antibiotika und Riboschaltern. a) Strukturen von Neomycin (NEO), Ribostamycin (RIO) und Paromomycin (PAR). Die Ringnummern sind mit lateinischen Ziffern angegeben (I–IV), die 6′‐Gruppen sind lachsfarben hervorgehoben. b) Struktur der 2‐Fluoradenin (2FA)‐Base, die in dieser Studie als NMR‐Sonde verwendet wird. c) Sekundärstruktur des Riboschalters in der Apo‐Form (links) und in Gegenwart eines Liganden (rechts), wobei die 2FA‐markierten Positionen durch Kugeln gekennzeichnet sind. d) ^19^F Spektren des 2FA‐markierten Apo‐Riboschalters (oben) und des Riboschalters in der Gegenwart von NEO, RIO oder PAR. Die Skizzen auf der rechten Seite fassen wichtige chemischen Eigenschaften der drei Liganden zusammen: Ring I enthält eine NH_3_^+^‐Gruppe (NEO und RIO) oder eine OH‐Gruppe (PAR) und Ring IV ist entweder vorhanden (NEO und PAR) oder abwesend (RIO). L1, L2 und L3 zeigen die chemischen Verschiebungen der verschiedenen ligandengebundenen Zustände (siehe unten).

Wir untersuchen hier die Dynamik eines synthetischen RNA‐Riboschalters, der seine Struktur bei der Wechselwirkung mit Neomycin (NEO) oder den eng verwandten Liganden Ribostamycin (RIO) und Paromomycin (PAR) erheblich ändert (Abbildung 1a).^[21]^ Die Strukturen dieser Riboswitch‐Ligandenkomplexe ähneln sich sehr.[^22^, ^23^] Dies steht im Kontrast zu den Unterschieden in ihrer regulatorischen Wirksamkeit zur Unterdrückung der Translationsinitiation, die bei NEO am höchsten, für RIO geschwächt und für PAR völlig abwesend ist,^[21]^ obwohl NEO und PAR sich nur in einer einzigen funktionellen Gruppe unterscheiden. Interessanterweise wurde eine erhöhte Dynamik in den Liganden‐RNA Komplexen mit einer geringeren In vivo‐Effizienz in Verbindung gebracht.^[23]^

## Resultate und Diskussion

Um die Dynamik des Riboschalters zu beobachten, haben wir die RNA gleichzeitig an den Positionen 16, 17 und 24 mit 2‐Fluoradenin mittels in vitro‐Transkription markiert (Abbildung 1b, c).[^24^, ^25^] Im apo‐Zustand zeigt das ^19^F NMR‐Spektrum erwartungsgemäß drei starke Resonanzen, die wir durch Mutagenese zugeordnet haben (Abbildung 1d, Abbildung S1). In Anwesenheit von NEO, RIO oder PAR ist die A17 ^19^F‐Resonanz deutlich tieffeldverschoben (Abbildung 1d). Diese große chemische Verschiebung stimmt mit den bekannten Strukturen überein, bei denen die Nukleobase von A17 direkt auf den Liganden stapelt und als Klappe der ligandenbindenden Tasche fungiert.[^22^, ^23^] Interessant ist, dass in Gegenwart von sättigenden Mengen der NEO‐ und PAR‐Liganden (anhand von 1D NMR‐Spektren und CEST‐Experimente beurteilt) zwei Resonanzen für A17 beobachtet werden. Beide Resonanzen unterscheiden sich von der Resonanz im Apo‐Zustand und entsprechen somit zwei ligandengebundenen Zuständen (bezeichnet als Ligandenzustände L1 und L2; Abbildung 1d).

Dieser Befund offenbart, dass mindestens zwei strukturell unterschiedliche Konformationen in den an NEO und PAR gebundenen Riboschaltern vorhanden sind, die auf der NMR‐Zeitskala langsam austauschen, d. h. die Austauschrate *k*_ex,L1‐L2_=*k*_L1→L2_+*k*_L2→L1_ ist viel kleiner als der chemische Verschiebungunterschied Δ*ν*_L1L2_=|*ν*_L1_−*ν*_L2_|=Δ*ω*_L1L2_/2π. Da sowohl NEO als auch PAR eine Aminoglykosideinheit (Ring IV) besitzen, die in RIO fehlt, vermuten wir, dass diese Einheit in Bezug auf A17 zwei Konformationen annehmen kann, eine, in der sie sich in der Nähe von A17 befindet (die wir als Zustand L1 bezeichnen), und eine, in der sie von A17 entfernt (NEO oder PAR) oder nicht im Liganden vorhanden ist (RIO) (diesen Zustand bezeichnen wir als L2) (Abbildung 1d).

**Figure 2:**
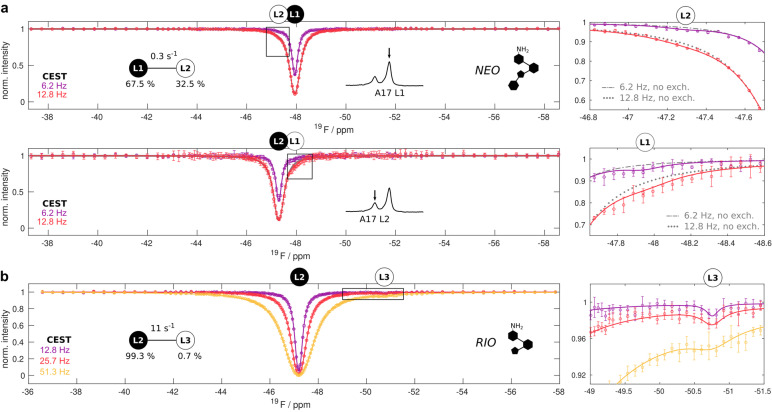
Langsamer Zwei‐Zustände‐Austausch in den NEO‐ und RIO‐gebundenen Riboschaltern. a) CEST‐Profile der L1‐ und L2‐Zustände des NEO‐gebundenen Riboschalters bei 303 K und bei B_1_‐Feldern von 6.2 Hz und 12.8 Hz. Die beste Anpassung an die Daten ist als durchgezogene Linie für einen Zwei‐Zustände‐Austauschprozess zwischen L1 und L2 dargestellt (Tabelle S2). Die analysierten Resonanzen (oben: L1, unten: L2) sind über den CEST‐Profilen und mit einem Pfeil in den Bereichen der angegebenen 1D‐Spektren angegeben (siehe auch Abbildung 1d). Die rechten Tafeln zeigen Vergrößerungen der CEST‐Profile, die in der linken Tafel durch Kästchen gekennzeichnet sind und bei denen die Daten von dem erwarteten CEST‐Profil bei fehlendem Austausch abweichen (kein Austausch in grau). b) CEST‐Profile des L2‐Zustands des RIO‐gebundenen Riboschalters bei 303 K und bei B_1_‐Feldern von 12.8 Hz, 25.7 Hz und 51.3 Hz. Die beste Anpassung zu den Daten ist als durchgezogene Linie für einen Zwei‐Zustände‐Austauschprozess zwischen L2 und L3 dargestellt (Tabelle S3). Die Resonanzpositionen von L2 und L3 sind oberhalb der CEST‐Profile angegeben. Die rechte Tafel zeigt eine Vergrößerung des CEST‐Profils, das in der linken Tafel durch einen Kasten gekennzeichnet ist. In allen Feldern sind die experimentellen Datenpunkte als Kreise mit Fehlerbalken dargestellt, die 1 Standardabweichung entsprechen.

Um die Bewegungen im NEO‐gesättigten Riboschalter zu studieren, haben wir ^19^F‐Relaxationsmessungen verwendet, die sich als wertvolle Ansätze zur Untersuchung niedrig besiedelter Zustände erwiesen haben.[^26^, ^27^, ^28^, ^29^, ^30^] Wir haben Chemische Austausch‐Sättigungsübertragungs‐(CEST) Experimente durchgeführt, bei denen die Magnetisierung bei variablen Frequenzabständen gesättigt und die verbleibende Magnetisierung detektiert wird.[^31^, ^32^] Wenn ein Kern dynamisch zwei Konformationen einnimmt, wird die selektive Störung eines Zustands (z. B. L1) die Magnetisierung im anderen Zustand (z. B. L2) beeinflussen. Eine sorgfältige Analyse dieser Daten für den an NEO gebundenen Riboschalter zeigte leicht verringerte Intensitätsniveaus der L1‐Resonanz bei Sättigung der L2‐Resonanz und umgekehrt (Abbildung 2a, rechts). Eine globale Parameterschätzung mit einem 2‐Zustände‐Austauschmodell ergab, basierend auf den CEST‐Profilen für L1 und L2, eine Austauschrate von *k*_ex_=0.29±0.03 s^−1^ und die Populationen *p*_L1_=67.5±3.9 % und *p*_L2_=32.5±3.9 % bei 303 K (Abbildung 2a).

Diese Populationen stimmen hervorragend mit den relativen Signalintensitäten der L1‐ und L2‐Zustände im 1D‐^19^F‐Spektrum überein (*I*_L1_=68 %, *I*_L2_=32 % für *I*_L1_+*I*_L2_=100 %). Zu beachten ist, dass die Austauschrate sehr langsam ist und wir daher nicht in der Lage waren, diesen Prozess mit Hilfe der 2D‐^19^F‐^19^F‐Longitudinalaustausch‐Spektroskopie (EXSY) nachzuweisen.^[33]^ Bei EXSY‐Experimenten bilden sich Kreuzsignale bei den Frequenzen (*ω*_L1_, *ω*_L2_) und (*ω*_L2_, *ω*_L1_) während einer Mischzeit, in der sich die Konformation der RNA ändern kann. Im Falle des mit NEO gesättigten Riboschalters sind die Relaxationsraten R1 der L1‐ und L2‐Resonanzen (≈2–3 s^−1^) jedoch schneller als die Austauschraten (0.29 s^−1^), was den Aufbau von EXSY‐Kreuzsignalen auf ein nachweisbares Niveau verhindert. Zusammenfassend lässt sich sagen, dass Ring IV in NEO langsam austauscht zwischen dem L1‐Zustand, in dem er direkt mit A17 wechselwirkt, und dem L2‐Zustand, in dem dieses Motiv von A17 entfernt ist.

Als nächstes untersuchten wir die Dynamik des RIO‐gebundenen Riboschalters, dem Ring IV fehlt und der daher nur die L2‐Resonanz aufweist (Abbildung 1d). Interessanterweise zeigen die CEST‐Profile dieser L2‐Resonanz (Abbildung 2b) einen angeregten Zustand, der bei −50.8 ppm resoniert (den wir als Zustand L3 bezeichnen), der in CEST‐Experimenten mit dem NEO‐Riboswitch nicht beobachtet wurde und der in den 1D NMR Spektren nicht unmittelbar sichtbar ist. Die CEST‐Daten lassen sich numerisch mit einem 2‐Zustände‐Austauschmodell mit einer L3‐Population von 0.7±1.3 % anpassen, welche mit dem zu 99.3±1.3 % besetzten L2‐Zustand mit einer Rate von *k*_ex_=11±15 s^−1^ austauscht, wobei die großen Unsicherheiten die kleine Population von L3 widerspiegeln. Um zusätzliche Erkenntnisse über den L2–L3 Austauschprozess[^34^, ^35^] zu gewinnen, haben wir CEST‐Datensätze bei 293 K, 298 K und 308 K aufgenommen (Abbildung S2). Bei 308 K stieg die L2–L3‐Austauschrate auf 21±17 s^−1^. Bei niedrigeren Temperaturen (293 und 298 K) war der L3‐Zustand nicht mehr zu beobachten. Zusammenfassend lässt sich sagen, dass der an RIO gebundene Riboschalter einen schwach besiedelten angeregten Zustand L3 einnimmt, in dem A17 eine chemische Verschiebung aufweist, die nahe an der chemischen Verschiebung von A17 im Apo‐Riboschalter liegt. Daraus schließen wir, dass die Klappen‐Nukleobase A17 im L3‐Zustand partiell vom Liganden dissoziiert.

**Figure 3:**
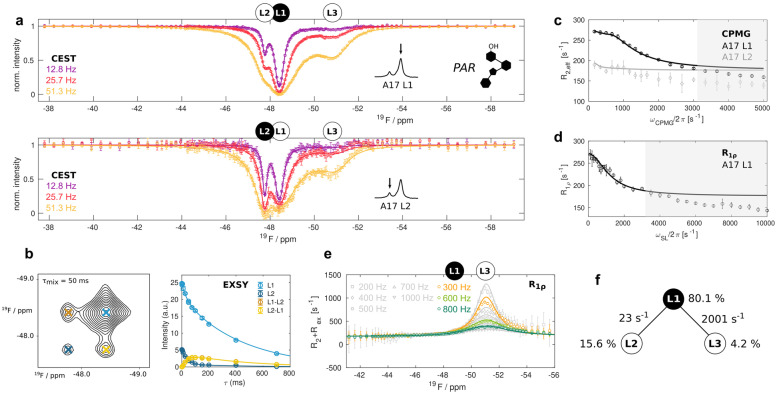
Drei‐Zustände‐Austausch im PAR‐gebundenen Riboschalter. a) CEST‐Profile aus der Analyse der L1‐ (oben) und L2‐Resonanzen (unten). b) Beispielhaftes [^19^F‐^19^F]‐EXSY‐Spektrum für eine Mischzeit von 50 ms und die Abhängigkeit der Intensitäten der Auto‐ (L1, L2) und Kreuzsignale (L1–L2, L2–L1) von der EXSY‐Mischzeit. c) CPMG‐RD‐Profile von L1 und L2. d) R_1ρ_‐RD‐Profil von L1 bei Resonanz. Die grau schattierten Bereiche in (c) und (d) wurden bei der Anpassung nicht verwendet und zeigen zusätzliche schnelle μs‐Zeitskalenbewegungen an, die hier nicht analysiert werden. e) R_2_+R_ex_‐Beiträge, abgeleitet aus “off‐Resonanz” R_1ρ_ RD‐Experimenten. Alle Daten in (a) bis (e) wurden bei 303 K und 11.7 T aufgezeichnet. Die Datenpunkte sind als offene Kreise mit Fehlerbalken dargestellt, die 1 Standardabweichung entsprechen, die globale Anpassung mit einem Drei‐Zustände‐Austauschmodell ist in allen Feldern als durchgezogene Linie dargestellt. f) Anpassungsparameter für das lineare L2–L1–L3‐Modell (Tabelle S6).

Im PAR‐Liganden (Abbildung 1a, d) enthält der Ring I, der direkt mit der A17‐Phosphatgruppe wechselwirkt, eine ‐OH‐Gruppe anstelle einer ‐NH_3_^+^‐Gruppe, wie sie in den NEO‐ und RIO‐Liganden vorhanden ist. Der PAR‐gebundene Riboschalter nimmt eine Struktur an, die insgesamt identisch ist mit dem RIO‐gebundenen Aptamer,[^22^, ^23^] allerdings keine stabilen A17 : Ligand‐ und A17 : C6‐Stapelwechselwirkungen^[23]^ aufweist, und nicht in der Lage ist, die ribosomale Abtastung sterisch zu blockieren. Um die molekulare Grundlage dieses funktionellen Unterschieds zu beleuchten, haben wir CEST‐Daten des PAR‐gesättigten Riboschalters bei 303 K aufgenommen (Abbildung 3a). Diese Daten zeigen deutlich, dass die Zustände L1, L2 und L3 gleichzeitig vorhanden sind. Ausgehend von 2D‐EXSY‐Experimenten (Abbildung 3b) schlussfolgern wir, dass die L1‐ und L2‐Zustände direkt miteinander austauschen, und zwar mit einer Rate in der Größenordnung von 25 s^−1^, d. h. zwei Größenordnungen schneller als beim L1–L2‐Austausch im Fall des NEO‐gebundenen Riboschalters. Basierend auf ersten Parameterschätzungen der CEST‐Daten liegen die Austauschraten zwischen den Zuständen L1/L2 und Zustand L3 bei 1500–2500 s^−1^. Austauschraten in diesem Bereich können mithilfe von CPMG‐Relaxations‐Dispersionsexperimenten (RD) genau quantifiziert werden.[^29^, ^34^] In diesen Experimenten wird die austauschinduzierte Linienverbreiterung in NMR‐Resonanzen durch eine variable Anzahl von Refokussierungspulsen unterdrückt. In unserem Fall beobachten wir eindeutig ein CPMG‐RD‐Profil für die L1 Resonanz (Abbildung 3c). Dieses RD‐Profil kann nicht direkt aus dem Austauschprozess zwischen L1 und L2 stammen, da dieser Prozess zu langsam ist, um in den CPMG‐Experimenten beobachtet zu werden. Daraus schließen wir schließen wir, dass der Zustand L1 direkt mit dem Zustand L3 austauscht.

Um weitere Einblicke in den L1↔L3‐Austauschprozess zu erhalten, haben wir “on‐Resonanz” und “off‐Resonanz” R_1ρ_‐RD‐Profile für den L1 Zustand aufgezeichnet, wobei die Relaxationsrate einer “spin‐locked” Resonanz als Funktion der “Spin‐Lock”‐Feldstärke bzw. des “Spin‐Lock”‐Offsets gemessen wird.^[29]^ Wir beobachten eindeutig einen Austausch in den L3‐Zustand (Abbildung 3d, e).

Um die Populationen und Raten für den Austauschprozess mit drei Zuständen (L1, L2, L3) im PAR‐gesättigten Riboschalter zu ermitteln, haben wir eine globale Parameterschätzung der CEST‐, EXSY‐, CPMG‐ sowie der On‐ und Off‐Resonanz R_1ρ_ RD Daten durchgeführt. Die Analyse ergab, dass ein lineares Modell, bei dem L2 und L3 nicht ausgetauscht werden, statistisch einem vollständigen Modell vorzuziehen ist, bei dem alle Zustände direkt miteinander austauschen (siehe Methoden). Zu beachten ist, dass das niedrige CPMG RD Amplitudenprofil für L2 (Abbildung 3c), durch den schnellen Austausch zwischen L1 und L3 aufgrund des langsamen Austauschs zwischen L1 und L2 verursacht wird. Die lineare Ausgleichsrechnung zeigt, dass bei 303 K die Populationen von L1, L2 und L3 80.1±0.1 %, 15.6±0.1 % bzw. 4.2±0.1 % betragen, und die Austauschraten L1↔L2 und L1↔L3 bei 23±0.4 s^−1^ und 2001±13 s^−1^ liegen (Abbildung 3f, Tabelle S6). Die Unterschiede in den L1↔L2‐Austauschraten zwischen den an PAR und NEO gebundenen Liganden implizieren, dass zwei entfernte Stellen im Liganden (die −NH_3_^+^/−OH Gruppe in Ring I und Ring IV) einen direkten Einfluss aufeinander haben und dass die Erkennung des Antibiotikums durch den Riboswitch komplexer ist als ein einfacher Bindungsprozess mit zwei Zuständen.

Um Informationen über die thermodynamischen Parameter des Austauschprozesses zu erhalten, der die drei Zustände im PAR‐gesättigten Riboschalter verbindet, haben wir ^19^F CEST, CPMG und EXSY Experimente bei 293 K, 298 K und 308 K durchgeführt (Abbildung S3). Für die Daten bei der jeweiligen Temperatur wurde global eine Parameterschätzung an das lineare 3‐Zustände‐Austauschmodell, das wir bei 303 K aufgestellt hatten, durchgeführt. Für die ermittelten Austauschparameter wurde anschließend mithilfe der Arrhenius‐ und Eyring‐Gleichungen eine Ausgleichsrechnung vorgenommen (Abbildung S4). Die Austauschprozesse L1↔L2 und L1↔L3 zeigen einen Enthalpie‐Entropie‐Ausgleich und sind entropisch begünstigt (Tabelle S9). Dies deutet darauf hin, dass die L2 und L3‐Zustände weniger geordnet sind als der L1‐Zustand, in Übereinstimmung mit einer Dissoziation von Ring IV von der RNA (L2) und einer Öffnung der A17‐Nukleobase (L3).

Die obigen Daten zeigen, dass es möglich ist, genaue Austauschparameter aus ^19^F‐Experimenten für ein System zu extrahieren, das drei strukturell unterschiedliche Zustände einnimmt. Dies veranlasste uns dazu, zu testen, ob auch andere 3‐Zustände‐Austauschprozesse analysiert werden können. Zu diesem Zweck erweiterten wir das 2‐Zustände‐System des RIO‐gesättigten Riboschalters (Abbildung 2b) auf ein 3‐Zustände‐System, indem wir eine Probe herstellten, in der der Riboschalter nicht vollständig mit RIO gesättigt ist. In diesem System entsprechen zwei Zustände den oben beschriebenen RIO‐gebundenen Riboschalter Konformationen (L2 und L3), und ein Zustand entspricht dem schwach besetzten und daher NMR‐unsichtbaren Apo‐Riboschalter (A). Wir nahmen ^19^F‐CEST‐Experimente für die A17 Resonanz bei 293 K, 298 K, 303 K und 308 K auf (Abbildung S5). Die Population des Apo‐Riboschalters (A) ist im CEST‐Profil des L2‐Zustands deutlich sichtbar. Darüber hinaus ist bei 303 K und 308 K der L3‐Zustand deutlich sichtbar, in Übereinstimmung mit den Daten, die mit dem Riboschalter aufgezeichnet wurden, der mit RIO gesättigt war (Abbildung 2b, Abbildung S2). Anschließend führten wir für die Daten bei 293 K und 298 K eine Parameterschätzung anhand eines 2‐Zustände‐Modells (A↔L2) und für die Daten bei 303 K und 308 K eine Parameterschätzung anhand eines linearen 3‐Zustände‐Modells (A↔L2↔L3) durch. Im letzteren Fall ist anzumerken, dass Anpassungen mit einem komplexeren Dreiecksmodells (bei dem die Zustände L3 und A auch direkt austauschen können) die gleichen χ_ν_‐Werte ergaben, was die bekannte Schwierigkeit widerspiegelt, kinetische Mehrzustandsmodelle in Gegenwart eines langsamen Austauschs zu unterscheiden.[^7^, ^36^, ^37^] Bei 303 K erhielten wir Populationen von 1.6±0.1 % (Zustand A), 98.2±0.1 % (Zustand L2), bzw. 0.3±0.1 % (Zustand L3), sowie eine Austauschrate von 22±6 s^−1^ für den L2↔L3‐Austausch (Abbildung S5).

Wichtig ist, dass die relativen L2 und L3 Populationen sowie die L2↔L3‐Austauschrate mit den Daten übereinstimmen, die sich für den gesättigten RIO‐Riboswitch ergaben (Abbildung 2b). Für den Austausch zwischen den Zuständen L2 und A erhielten wir *k*_ex_=[L]*k*_on_+*k*_off_=88±8 s^−1^, woraus wir eine Dissoziationsrate für den RIO‐Liganden von ≈1.4 s^−1^ erhalten. Sowohl die Population des Apo‐Riboschalters (A), als auch die Austauschrate zwischen dem A‐ und L2‐Zustand nehmen mit der Temperatur zu (Abbildung S5), in Übereinstimmung mit einem Entropiezuwachs für den Übergangszustand des dissoziierenden Liganden.

**Figure 4:**
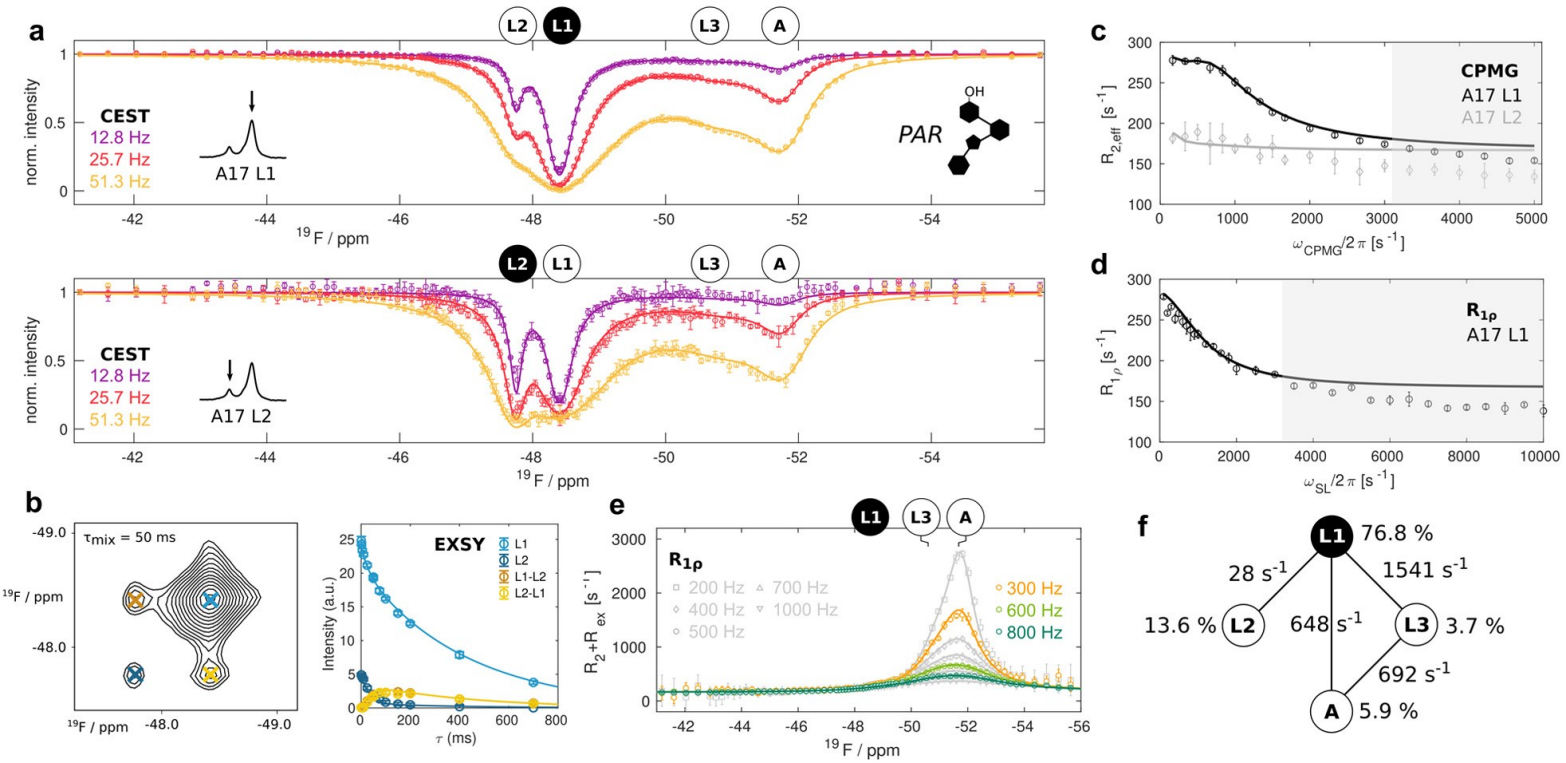
Ein Vier‐Zustände‐Austausch im ungesättigten PAR‐gebundenen Riboschalter. a) CEST‐Profile aus der Analyse der L1‐ (oben) und L2‐Resonanzen (unten). b) Exemplarisches [^19^F‐^19^F] EXSY‐Spektrum für eine Mischzeit von 50 ms und die Abhängigkeit der Intensitäten der Auto‐ (L1, L2) und Kreuzsignale (L1–L2, L2–L1) von der EXSY‐Mischzeit. c) CPMG RD‐Profile von L1 und L2. c) CPMG RD‐Profil von L1 und L2. d) R_1ρ_‐RD‐Profil bei Resonanz. Die grau schattierten Bereiche in (c) und (d) werden in der Anpassung nicht verwendet und zeigen zusätzliche schnelle μs‐Zeitskalenbewegungen an, die hier nicht analysiert werden. e) R_2_+R_ex_‐Beiträge, abgeleitet aus “Off‐Resonanz” R_1ρ_ RD‐Experimenten. Alle Daten in (a) bis (e) wurden bei 303 K und 11.7 T aufgezeichnet. Die Datenpunkte sind als offene Kreise mit Fehlerbalken dargestellt, die 1 Standardabweichung entsprechen, die globale Anpassung mit einem Vier‐Zustands‐Austauschmodell ist in allen Feldern als durchgezogene Linie dargestellt. f) Anpassungsparameter für das Austauschmodell (Tabelle S7).

**Figure 5:**
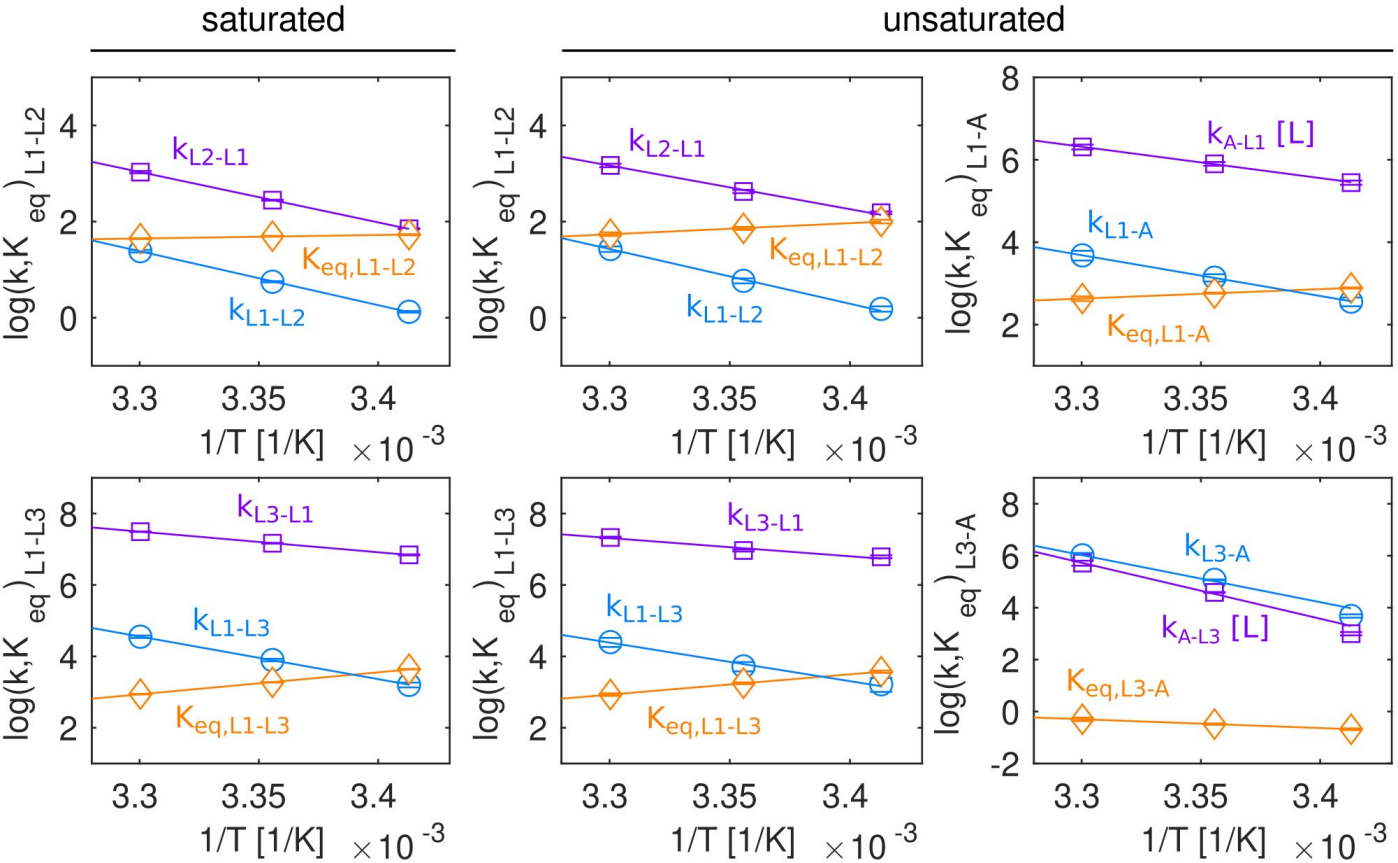
Thermodynamische Analyse der Dynamik im PAR‐gebundenen Riboschalter. Vergleich der Temperaturabhängigkeit der Vorwärtsraten, Rückwärtsraten und Gleichgewichtskonstanten zwischen verschiedenen Zuständen des Neomycin‐Riboschalters, der an PAR gebunden ist, bei sättigenden (links; Abbildung 3, Abbildung S5, Tabelle S9) und nicht‐sättigenden (rechts; Abbildung 4, Abbildung S9, Tabelle S9) Bedingungen. Die Datenpunkte bei 308 K wurden bei der Anpassung ausgelassen (siehe ergänzendes Material) und sind nicht dargestellt. Zu beachten ist, dass die Bindung des Liganden an den Riboswitch ein bimolekularer Prozess ist, der von der Konzentration des freien Liganden abhängt, wobei z. B. *k*_ex, L3‐A_=*k*_off_+[L]*k*_on_=*k*_L3‐A_+[L]*k*_A‐L3_.

Schließlich weiteten wir unsere Analyse auf ein System aus, in dem der Riboswitch nur teilweise mit PAR gesättigt ist und somit insgesamt vier strukturell unterschiedliche Zustände aufweist: den Apo‐Riboschalter (A) und die drei PAR‐gebundenen Zustände (L1, L2 und L3). Wir zeichneten einen vollständigen Satz von ^19^F‐Relaxationsexperimenten (CEST, CPMG, On‐Resonanz R_1ρ_, Off‐Resonanz R_1ρ_, EXSY) für die A17 Resonanzen bei 303 K auf (Abbildung 4). Bei den CEST‐Experimenten wurde die Resonanz des Apo‐Riboschalters in der Sättigung der Resonanzen der Zustände L1 und L2 beobachtet. Damit übereinstimmend fanden wir eine deutliche Zunahme der Relaxationsrate im rotierenden Bezugssystem R_1ρ_ und ihres (R_2_+R_ex_)‐Beitrags bei dem “Offset” der Resonanz des freien Riboschalters. Im Gegensatz dazu ähneln die CPMG‐ und die On‐Resonanz R_1ρ_ RD Experimente denjenigen vom PAR‐gesättigten Riboschalters. Dies deutet darauf hin, dass die Bindung von PAR in einem Austauschregime stattfindet, das zu langsam ist, um in diesen RD‐Experimenten nachweisbar zu sein. In Übereinstimmung damit beobachten wir schwache EXSY‐Kreuzpeaks zwischen den Zuständen A und L2 (Abbildung S6). Wir passten verschiedene 4‐ Zustände‐Austauschmodellen an den vollständigen Satz von Relaxationsdaten an und stellten fest, dass das wahrscheinlichste der 38 möglichen Modellen dasjenige ist, bei dem ein direkter Austausch zwischen L1 und L2, L1 und L3, L1 und A sowie zwischen L3 und A berücksichtigt wird (4 Kanten; Abbildung S10, Tabelle S8, ergänzende Methoden). Eine globale Anpassung aller Relaxationsdaten ergab, dass die Zustände A, L1, L2 und L3 zu 5.9±0.2, 76.8±0.6, 13.6±0.6 bzw. 3.7±0.2 % besetzt sind (Abbildung 4f, Tabelle S9). Die Austauschraten zwischen L1, L2 und L3 (Abbildung 3) sind erwartungsgemäß durch das Vorhandensein des Apo‐Zustands weitgehend unbeeinflusst. Weiter stellten wir fest, dass der freie Riboschalter (A) mit den Zuständen L1 (648±33 s^−1^) und L3 (691±76 s^−1^) mit ähnlichen Raten austauscht (*k*_ex_). Die Dissoziationsrate des PAR‐Liganden ist somit 10 (vom Zustand L1) bis 100 (vom Zustand L3) mal schneller als die Dissoziationsrate für den RIO‐Liganden (Abbildung S5). Dies ist im Einklang mit ITC‐Experimenten, die zeigen, dass PAR schwächer an den 2FA‐markierten Riboswitch bindet als RIO (Abbildung S7). Theoretisch ist es möglich, Dissoziationskonstanten (*K*_D_=[freie RNA][freier Ligand]/[RNA : Ligand]) aus den Populationen zu erhalten, die wir hier abgeleitet haben. Allerdings führen selbst kleine Unsicherheiten bei den absoluten RNA‐ und Ligandenkonzentrationen in den NMR‐Proben zu sehr großen Unsicherheiten in den extrahierten Affinitäten, was eine sinnvolle Extraktion von *K*_D_‐Werten aus den NMR abgeleiteten Populationen verhindert. Um thermodynamische Informationen für den Vier‐Zustands‐Austauschprozess zu extrahieren, nahmen wir reduzierte Sätze von Relaxationsdaten (CEST, CPMG, EXSY) bei 293 K, 298 K und 308 K (Abbildung S8) auf und verwendeten das aus den Daten bei 303 K abgeleitete Modell zur Parameterschätzung für diese Daten. Ebenso wie im RIO‐Komplex zeigt ein Arrhenius/Eyring‐Modell, dass die PAR‐Bindung mit einem großen entropischen Verlust verbunden ist (Abbildung 5, Abbildung S9, Tabelle S9). Darüber hinaus konnten wir unsere vorherige Erkenntnis bestätigen, dass die Besetzung angeregter, an den Liganden gebundener Zustände entropisch begünstigt ist und eine Enthalpie‐Entropie‐Kompensation aufweist.

## Zusammenfassung

**Figure 6:**
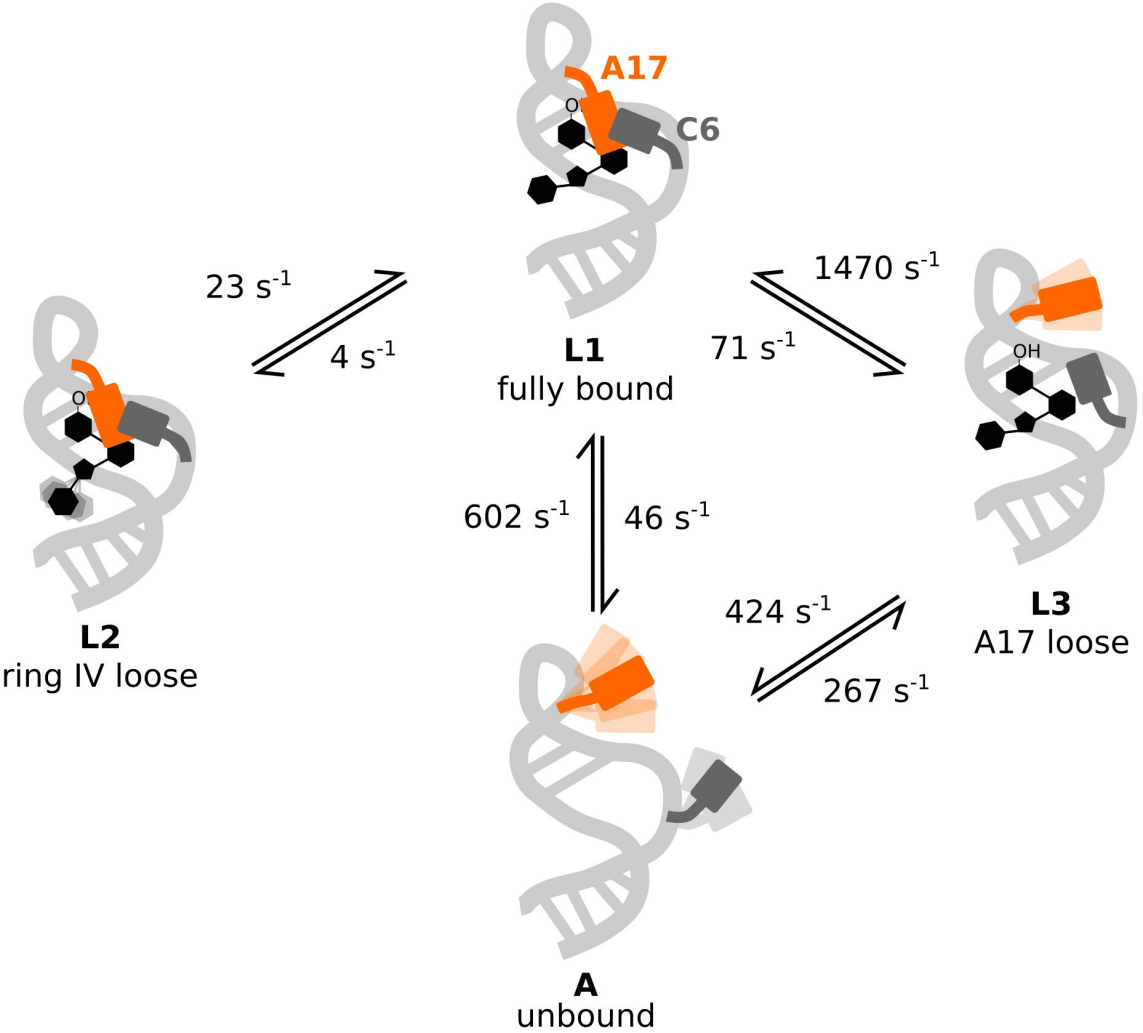
Modell der Austauschprozesse im ungesättigten PAR‐gebundenen Neomycin‐bindenden Riboschalter. Im vollständig gebundenen Zustand (L1) interagiert Ring IV des Liganden mit U18 und G19, während Ring I an A17 andockt (orange). Letztere Wechselwirkung geht mit der Bildung einer C6 (dunkelgrau) : A17 “Büroklammer”‐ Stapelwechselwirkung einher.^[38]^ Aus diesem Zustand L1 kann Ring IV des Liganden dissoziieren, sodass der “lockere Ring‐IV‐Zustand” (L2) gebildet wird. Dieser Zustand ist auch im RIO‐gebundenen Riboschalter vorhanden (dem der Ring IV fehlt), für den eine stabile A17 : C6‐Stapelung experimentell verifiziert wurde.^[23]^ Alternativ kann A17 aus dem L1‐Zustand herausklappen (z. B. in Gegenwart von PAR), sodass der “lockere A17‐Zustand” gebildet wird.

Zusammengefasst nutzen wir hier komplementäre ^19^F NMR Relaxationsexperimente, um einen Einblick in die komplexen dynamischen Konformationsgleichgewichte des Neomycin‐bindenden Riboschalters im Komplex mit den Liganden NEO, RIO und PAR zu erhalten. Unsere Ergebnisse zeigen einen modularen Einfluss der Beschaffenheit des Aminoglykosids auf die Konformationsenergielandschaft der ligandengebundenen Riboschalter (Abbildung 6): Erstens führt die Anwesenheit von Ring IV (in NEO und PAR) zu einem Konformationsgleichgewicht zwischen zwei langsam austauschenden Zuständen (L1 und L2). Ring IV ist in der NMR‐Lösungsstruktur des PAR‐gebundenen Riboschalters strukturell nicht definiert, MD Simulationen deuten jedoch darauf hin, dass dieser Ring die Phosphatgruppen von U18 und G19 kontaktieren kann.^[38]^ Die L1‐ und L2‐Zustände stellen somit Sub‐Ensembles dar, in denen Ring IV entweder an das RNA‐Rückgrat angedockt (L1) oder lose (L2) ist. Zweitens bestimmt die 6′‐Gruppe von Ring I (−NH_3_^+^ in NEO und RIO, −OH in PAR) die relative Stabilität der A17 Klappe, die entweder geschlossen (L1 und L2) oder offen (L3) sein kann. In Übereinstimmung mit unseren NMR‐Daten berichten frühere MD‐Simulationen, dass A17 ausklappen kann, wenn PAR an den Riboswitch gebunden ist. In diesen MD‐Simulationen wurde das Ausklappen von A17 für RIO‐gebundenen Riboschaltern nicht beobachtet,^[39]^ was mit der Seltenheit dieses Ereignisses in Gegenwart von RIO übereinstimmt (L3‐Population <1 %). Experimentell wird das Ausklappen der A17‐Nukleobase außerdem durch die chemische Verschiebung der L3‐Resonanz gestützt (Abbildung 1d) die in der Nähe der Resonanz des freien Zustands (A) liegt, sowie durch den großen entropischen Beitrag zum L1–L3‐Austauschprozess (Abbildung 5).

Unsere Daten zeigen, dass die L1‐ und L2‐Konformationen beide funktional sind. da der NEO‐gebundene Riboschalter, der diese beiden Zustände enthält, in der Lage ist, die Translation vollständig zu hemmen. Die geringere Effizienz der Blockade der Translationsinitiation im RIO‐gebundenen Riboschalter korreliert gut mit dem Vorhandensein des Zustands L3, der strukturell näher am Apo‐Riboschalter liegt als die Zustände L1 und L2. In der Anwesenheit von PAR ist der Zustand L3 noch stärker ausgeprägt und folglich ist PAR nicht in der Lage, die Translation zu blockieren. Es sollte jedoch beachtet werden, dass die In vivo‐Ribosomenblockade ein hochkomplexer Prozess ist, an dem verschiedene zelluläre Faktoren beteiligt sind. Daher ist es noch nicht möglich, die unterschiedlichen Ligandenbindungsraten oder die verschiedenen strukturellen Zustände eindeutig mit der Funktion zu korrelieren.

Zusammenfassend lässt sich sagen, dass unsere Ergebnisse ein Licht auf die komplexe Dynamik von Liganden‐gebundenen Neomycin‐sensitiven Riboschaltern werfen und das Potenzial der ^19^F NMR zum Nachweis und zur quantitativen Untersuchung von Systemen mit komplexen Austausch‐Topologien unterstreichen. Dieser ^19^F‐Ansatz ergänzt die NMR‐Experimente zur Untersuchung der Konformationsdynamik in Nukleinsäuren, welche z. B. ^1^H‐, ^13^C‐ und ^15^N‐Isotope als Reporter verwenden.[^40^, ^41^, ^42^, ^43^] Die ^19^F‐Methodik wird hier beispielhaft für einen nicht‐trivialen 4‐Zustands‐Austauschmechanismus in einem synthetischen Riboschalter gezeigt,^[44]^ ist aber auch für andere RNAs, Proteine und deren funktionelle Komplexe anwendbar. Die Empfindlichkeit des Ansatzes ist beispiellos, da Populationen von nur 0.3 % und Raten von 0.3 s^−1^ nachgewiesen werden können. Auf dieser Grundlage erwarten wir, dass künftige Studien an einem breiten Spektrum von Biomolekülen zur Identifikation zahlreicher unsichtbarer und niedrig besiedelte Konformationen führen werden.

## Interessenkonflikt

CK ist Berater von Innotope und hält eine Beteiligung an Innotope, einem Unternehmen, das Produkte zur Markierung stabiler RNA‐Isotope anbietet. Die übrigen Autoren erklären, dass sie keine konkurrierenden Interessen haben.

1

## Supporting information

As a service to our authors and readers, this journal provides supporting information supplied by the authors. Such materials are peer reviewed and may be re‐organized for online delivery, but are not copy‐edited or typeset. Technical support issues arising from supporting information (other than missing files) should be addressed to the authors.

Supporting Information

## Data Availability

Die Daten, die die Ergebnisse dieser Studie unterstützen, sind auf begründete Anfrage beim Autor erhältlich.
